# Spent Mushroom Substrate Reused as Organic Fertilizer Enhances Lettuce (*Lactuca sativa* L.) Quality and Soil Nutrients: Insights from Physicochemical and Microbiome Analyses

**DOI:** 10.3390/microorganisms14050985

**Published:** 2026-04-28

**Authors:** Lin Yang, Zhengpeng Li, Shiwei Wei, Qin Dong, Lei Zha, Changxia Yu, Yan Zhao

**Affiliations:** 1 Institute of Edible Fungi, Shanghai Academy of Agricultural Sciences, Shanghai 201403, China; ylin_jade@163.com (L.Y.); lizp_ln@126.com (Z.L.); maomao88719@163.com (Q.D.); zhalei@saas.sh.cn (L.Z.); 2 Shanghai Agrobiological Gene Center, Shanghai 201106, China; wsw@sagc.org.cn

**Keywords:** lettuce, spent mushroom substrate, soil, microbial community, recycle

## Abstract

Returning spent mushroom substrate (SMS) to the field is an effective way to dispose of it. However, given the substantial nutrient consumption associated with *Volvariella volvacea* SMS, their effects on soil properties and crop performance warrant further investigation. By analyzing the effects of three different application rates of SMS on soil nutrients and lettuce (*Lactuca sativa* L.) quality, the results showed that the group with 1.5 kg m^−2^ SMS addition improved the total nitrogen (+21.2%), and organic content (+27.9%) in soil, and it demonstrated particularly outstanding performance in enhancing the survival rate (+21.9%), average weight (+71.7%), chlorophyll content (+45.6%), and total phenolic content (+25.2%) of lettuce. By comparing the soil microbial communities in the control group, the SMS (1.5 kg m^−2^) treatment group, and the organic fertilizer treatment group, it was found that they were mainly composed of Group S1, S2, and S3 microorganisms, respectively. The microbial community evenness in the treatment groups was greater than that in the control group. Furthermore, the results also revealed that the microbial conversion efficiency of nitrogen and phosphorus in the SMS treatment group was higher than the control group, which promoted nutrient cycling and improved the quality of lettuce. Our analysis provides an environmentally friendly way for *Volvariella volvacea* SMS disposal.

## 1. Introduction

Lettuce is rich in various nutrients, including vitamin C, total phenolics, and chlorophyll [1]. Owing to its suitability for raw consumption, it retains more comprehensive nutrients than thermally processed foods, making it a key ingredient in salads [2]. In modern society, as living standards continue to improve, maintaining good health has become a common goal [3]. Therefore, lettuce meets the demand for a healthy diet, leading to its growing market demand. In addition, lettuce is a cool-season crop with a short vegetation period and can be cultivated throughout the year, which benefits farmers [4]. To increase vegetable yields, farmers often apply chemical fertilizers in amounts that far exceed the crops’ requirements. Excess fertilizer in the soil may cause potential damage to soil, such as soil acidification, soil compaction and a decrease in soil microbial diversity [5]. Moreover, surplus fertilizers can be carried into river systems by rainfall, adversely affecting aquatic environments—for instance, resulting in water eutrophication [6]. To solve these problems, organic fertilizer is a good alternative. There are many types of organic fertilizers, such as animal manure, rice straw, and activated sludge [7,8,9]. Spent mushroom substrate (SMS) can also be used as fertilizer in the field [10].

SMS is a waste product generated from the mushroom industry. As reported, for each kilogram of mushrooms produced, 3 to 5 kg of SMS was generated [11]. Globally, approximately 60 million tons of SMS are produced every year [11]. The low utilization rate of SMS leads not only to the wastage of valuable agricultural resources but also to serious environmental issues, such as soil contamination and air and water pollution [12]. Although SMS is classified as agricultural waste, it is rich in minerals such as N, P, and K, which can provide nourishment for plants [13]. It also contains components such as protein, fat, and fiber, as well as bioactive substances such as polysaccharides, acids, enzymes, and phenolics, which can supply nutrients to microorganisms in soil [14]. Furthermore, SMS has a low bulk density and high porosity, making it a material with favorable properties for improving soil [15]. Thus, returning SMS to fields can not only solve the problem of SMS disposal but also improve the soil quality. Several reports have investigated the application of SMS as an organic fertilizer to improve soil. Li et al. [10] investigated changes in soil organic matter and microbial diversity after ten years of applying SMS of *Agaricus bisporus* in paddy fields, whereas Alves et al. [16] examined the effects of SMS on soil mineral contents and maize yields.

*Volvariella volvacea*, a type of mushroom with a short growth cycle, is highly valued for its delicious taste and nutritional richness, leading to its increasing cultivation [11]. Unlike other edible fungi cultivation process, to reduce production costs, certain types of spent mushroom substrates, such as those derived from *Hericium erinaceus*, are supplemented with a portion of rice straw and used in combination with low-cost cotton waste for the cultivation of *V. volvacea* [11]. However, the disposal of its SMS poses a challenge to sustainable development. The application of this SMS to agricultural fields as organic fertilizer constitutes an effective disposal method. However, given that the substrate employed for *V. volvacea* cultivation has undergone multiple cycles of utilization, the extent to which its residual nutritional quality remains sufficient to enhance soil fertility and improve vegetable quality warrants further investigation. To solve this problem, this study used lettuce as a model crop and investigated the application of *V. volvacea* SMSs in the field and compared this with commercially available organic fertilizer (cow manure). The objectives are to (1) examine the effects of SMS on lettuce growth and quality; (2) assess its impact on soil nutrients and soil microbial communities; and (3) elucidate the underlying microbial mechanisms through which SMS application influences lettuce growth.

## 2. Materials and Methods

### 2.1. Experimental Design

This test period was from 1 March 2023 to 28 April 2023, in an open test field at Jinshan Experimental Station of Shanghai Agrobiological Gene Center (30°48′ N, 121°10′ E) in Shanghai, China. Five plots of land were used in this research, and each plot was 4 × 5 m^2^ in size (Figure S1). The SMS and cow manure were applied to the soil using a tillage applicator (Shandong Changnong Machinery Co., Ltd., Dezhou, China) on 1 March 2023.

SMS is produced after the mushrooms are harvested and is fermented for two months, while the organic fertilizer is made from composted cow dung, and they all were obtained from the Zhuanghang Experimental Station of the Shanghai Academy of Agricultural Sciences (Shanghai, China). The SMS contained 2.01 g kg^−1^ total P, 13.29 g kg^−1^ total N, 7.28 g kg^−1^ total K and 270.21 g kg^−1^ organic matter. The organic fertilizer contained 1.51 g kg^−1^ total P, 16 g kg^−1^ total N, 9.95 kg^−1^ total K and 150.21 g kg^−1^ organic matter. The soil in the field contained 0.87 g kg^−1^ total P, 1.23 g kg^−1^ total N, 19.59 g kg^−1^ total K and 29 g kg^−1^ organic matter. To ensure the accuracy of the experiment, no additional nutrients were added except for the above substances.

On the basis of the popular genotype in Shanghai, and the rich nutrients, *Lactuca sativa* L. (Hunong No.1) was selected as the plant material in this research. The seedlings were provided by the Jinshan Experimental Station of Shanghai Agrobiological Gene Center (Shanghai, China). Lettuce was planted approximately 30 cm apart, and it was harvested at 59 days after all the manure was added to the lettuce.

As shown in Figure S1, five plots of equal size were selected in a random design: control group (CK): no treatment. Four plots were given SMS (0.75 kg m^−2^, 1.5 kg m^−2^, and 2.25 kg m^−2^, which are referred to as A, B, and C, respectively) to examine the influence of SMS on the lettuce quality. The SMS application rate referred to the study reported by the Muchena et al. [17]. The last plot was given organic fertilizer (5 kg m^−2^).

According to S-shaped sampling method, ten replicate soil core samples were taken from each test plot to a depth of 10 cm and then mixed thoroughly. At the same time, ten lettuces were also harvested for the crop properties tests. The sampling times were 0, 35 and 59 days, which were labeled M1, M2 and M3, respectively.

### 2.2. Chemical Properties of Soil

Soil samples were air-dried indoors, then crushed, sieved, and used for chemical properties testing. As reported, the total nitrogen was measured by the Kjeldahl method [18]. Total phosphorus and total potassium were measured according to the methods described by GB 9837—88 and NY/T 87-1988, respectively. Briefly, 0.2 g of dried soil was digested with 2 g NaOH at 640 °C for 15 min and then the solution was transferred to a volumetric flask and diluted to the mark; the total potassium was determined using the molybdenum blue colorimetric test (280FS-AA, Agilent, Santa Clara, CA, USA), whose detection line was 10^−12^ g. Total phosphorus was tested using spectrophotometry (AquaMate 8100, Thermo Fisher Scientific, Waltham, MA, USA).

### 2.3. Lettuce Growth, Harvest and Measurements

The average heights (cm), weights (g) and survival rates (%) of lettuce plants were determined.

Leaf samples from lettuce were rapidly frozen with liquid nitrogen and then stored at −80 °C for biochemical property analyses in lettuce leaves, including the chlorophyll content, soluble sugar content, soluble protein content, ascorbic acid content and total phenol content. To determine the amount of chlorophyll, 0.2 g of fresh leaf tissue was obtained from the lettuce after harvesting and extracted using 95% ethanol. The chlorophyll contents include the contents of chlorophyll a and chlorophyll b, whose concentrations are proportional to the absorbance values of ultraviolet light at 645 nm and 663 nm, respectively [19]. The total soluble sugar and soluble protein contents in the leaves were analyzed by electron colorimetry and Coomassie brilliant blue G-250 staining, respectively [20,21]. Ascorbic acid reduces ferric ions (Fe^3+^) to ferrous ions (Fe^2+^), which can react with bathophenanthroline to form a red chelate with an absorption peak at 534 nm. The absorption values indicate the ascorbic acid concentrations [22]. The total phenolic content was also determined using a spectrophotometric method. In this assay, phenolic acids were extracted from the leaves with methanol, and were then quantified by a UV‒Visible spectrophotometer [23].

### 2.4. Total DNA Extraction and Testing

Soil microbial community was analyzed in this study. At the harvest of the CK, B and D groups, three soil samples from each pot were collected and mixed thoroughly and then frozen by liquid nitrogen and stored at −80 °C for DNA extraction and quantification. Total genomic DNA was extracted, and quality checks and determinations of DNA concentrations were conducted according to Li et al. [24].

The primers, 338F (5′-ACTCCTACGGGAGGCAGCA-3′) and 806R (5′-GGACTACHVGGGTWTCTAAT- 3′), were used to perform PCR amplification and MiSeq sequencing on the V3–V4 region of the bacterial 16S rRNA gene. The PCR amplification and quantification steps were conducted according to the methods of Li et al. [24]. After the individual quantification step, amplicons were pooled in equal amounts, and paired-end 2250 bp sequencing was performed using an Illumina NovaSeq platform (NovaSeq 6000) with a NovaSeq 6000 SP Reagent Kit (Illumina, San Diego, CA, USA) (500 cycles), or paired-end 2300 bp sequencing was performed using an Illumina MiSeq platform (NextSeq 1000/2000) with a MiSeq Reagent Kit (Illumina, San Diego, CA, USA) v3 at Shanghai Personal Biotechnology Co., Ltd. (Shanghai, China). The Phylogenetic Investigation of Communities by Reconstruction of Unobserved States (PICRUSt) pipeline was used to further analyze the 16S rRNA gene data of the soil samples to predict the microbial metabolic activities on the basis of the 16S rRNA gene data and the Kyoto Encyclopedia of Genes and Genomes (KEGG) database.

### 2.5. Statistical Analysis

Statistical analyses of the soil chemical properties and lettuce biochemical properties were conducted with GraphPad Prism software, version 9 (GraphPad, San Diego, CA, USA). Normality of data was assessed using the Shapiro–Wilk test. When normality of variance assumptions was satisfied, One-way ANOVA analysis was employed and then it was corrected using Tukey’s multiple comparisons test. Adjusted *p*-values are reported, and statistical significance was set at *p* < 0.05 after correction. Sequence data analyses and the redundancy analysis (RDA) were performed at Shanghai Personal Biotechnology Co., Ltd. (Shanghai, China). mainly using the QIIME2 (2024.5) and R packages (v4.3.3). The Kruskal–Wallis test was employed for the microbial species differential analysis, and Dunn’s method was used for the post-hoc analysis. The redundancy analysis (RDA) was performed by R packages (v4.3.3), and the significance of RDA analyses was determined using a Permutation Test.

The odds ratios were used to determine whether functional genes were enriched in the treated soil samples (Groups B and D) compared with that in the CK Group [25].

$$OR=\frac{y_{i}/(1-y_{i})}{y_{CK}/(1-y_{CK})}$$

where y*_i_* and y*_CK_* are the relative abundances of a metabolic category as reported by the KEGG database in the treated samples (Groups B and D) and the control group (Group CK), respectively.

### 2.6. Economic Costs Required for Increasing Lettuce Weight Calculation

The economic costs calculation was performed only calculate the SMS and organic fertilizer cost. This calculation uses the weight of lettuce in the control group as the baseline to determine, for each of the four treatment groups, the monetary cost of additives consumed per 1 g increase in lettuce weight. The detailed calculation process is shown in the File S1.

## 3. Results and Discussion

### 3.1. Soil Physicochemical Properties

**Figure 1:**
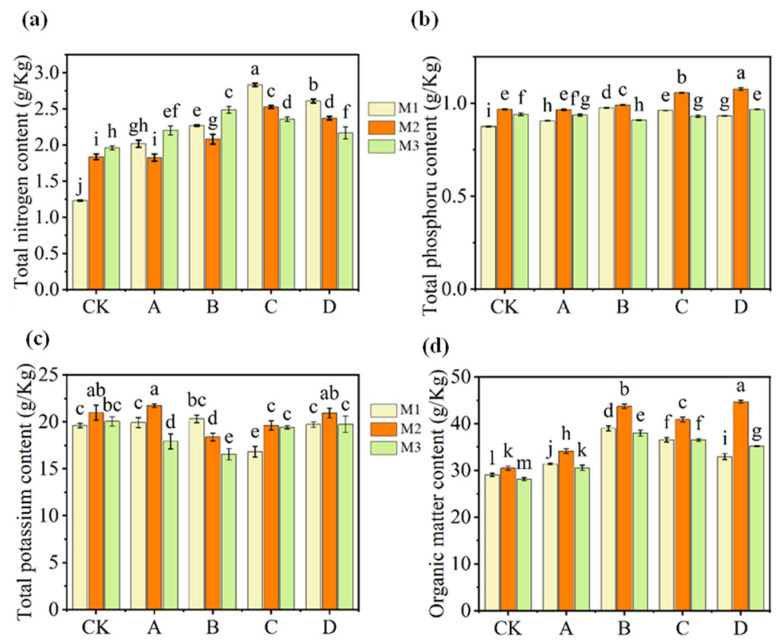
Total nitrogen (**a**), total phosphorus (**b**), total potassium (**c**) and organic matter contents (**d**) in the soils of the control group and different treatment groups during three phases: SMS and organic manure at first application (M1), lettuce growth at 35 days (M2), and lettuce harvest (M3). Error bars represent ± SEM of three replicates. Means with different lowercase letters in the same figure differ significantly (One-way ANOVA analysis, *p* < 0.05).

Figure 1a shows the total nitrogen changes, when SMS and organic fertilizer were firstly applied to the soil (M1 phase), compared with that in the control group (1.23 ± 0.01 g kg^−1^), the total nitrogen contents in the soil significantly increased in both the SMS treatment groups (Groups A (2.01 ± 0.05 g kg^−1^), B (2.27 ± 0.01 g kg^−1^) and C (2.83 ± 0.02 g kg^−1^)) and the organic fertilizer treatment group (2.61± 0.03 g kg^−1^ in Group D), with a gradual increase as the SMS application rate increased. One month after the lettuce was planted (M2 phase), the total nitrogen content in the control group soil increased, whereas these contents decreased in the treatment groups. When the lettuce was harvested (M3 phase), the nitrogen content continued to decline in the group with an SMS application rate of 2.25 kg m^−2^ and in the organic fertilizer group, whereas they increased in the other groups. Unlike the total nitrogen content, during the M1 phase, there were slight differences in the total phosphorus contents between the treatment groups (Groups A (0.91 g kg^−1^), B (0.98 g kg^−1^), C (0.96 g kg^−1^) and D (0.93 g kg^−1^)) and the control group (0.88 g kg^−1^), and all groups showed increases in the M2 phase—with the C and D groups exhibiting the greatest increases. Finally, in the M3 phase, the total phosphorus contents decreased across all groups (Figure 1b). As with the total phosphorus contents, the total potassium contents in the soil during the M1 phase differed little among the treatment groups and the control group, except for Group C; that is, SMS was 2.25 kg m^−2^. In the M2 phase, the contents increased in all the groups except for Group B (the SMS was 1.5 kg m^−2^), whereas in the M3 phase, the total potassium contents decreased across all the groups. The organic matter contents were also detected, and the results are shown in Figure 1d. Compared with the control group, the organic matter contents in the treatment groups increased, with Group B showing the highest content (38.98 ± 0.52 g kg^−1^). During the M2 phase, the organic matter contents continued to increase, reaching 43.73 ± 0.46 g kg^−1^ in Group C and 44.69 ± 0.32 g kg^−1^ in Group D. However, by the M3 phase, the contents decreased across all groups.

Among all the soil physicochemical properties, the addition of SMS and organic fertilizer improved the contents of nitrogen, phosphorus, potassium, and organic matter in the soil. As the lettuce grew, these nutrients were gradually released. However, by the time of lettuce harvest, their contents had decreased compared with the midterm levels. This phenomenon may be attributed to the following reason: over time, soil microorganisms decomposed SMS and organic fertilizer, releasing nutrients and leading to increased nutrient contents at midterm. These nutrients are subsequently absorbed and utilized by the lettuce, resulting in a decrease in their contents during the harvest phase [26].

### 3.2. Effects of SMS and Organic Fertilizer on Lettuce Growth and Yields

**Figure 2:**
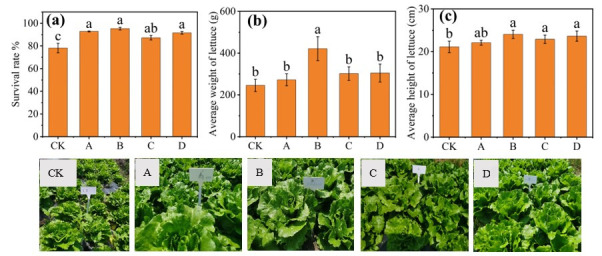
Effects of mushroom and organic manure on the growth of lettuce. The plant survival rates (**a**), average weights (**b**), and average heights (**c**) were measured when the lettuce was harvested. The data are presented as the means ± SDs (*n* = 10), Means with different lowercase letters (a–c) in the same figure differ significantly (One-way ANOVA analysis, *p* < 0.05). Pictures of the lettuce before harvest are shown.

The survival rates of lettuce are shown in Figure 2a. Compared with that in the control group, the survival rates of lettuce in all the treatment groups with SMS and organic fertilizer amendments significantly improved, with the greatest increase reaching 22%. At harvest (M3 phase), the average plant weight of the lettuce in Group B (SMS was 1.5 kg m^−2^) was the highest (421.4 ± 57.32 g), while that of the control group reached only 245.3 ± 29.42 g. The heights of the treated lettuce plants also exceeded those of the control, with Groups B and D showing the tallest plants (24.05 ± 0.97 cm and 23.62 ± 1.12 cm, respectively). Therefore, on the basis of these combined growth indicators, it can be concluded that the amendments can effectively increase lettuce yields, with Group B demonstrating particularly pronounced improvements.

### 3.3. Changes in Lettuce Biochemical Properties

**Figure 3:**
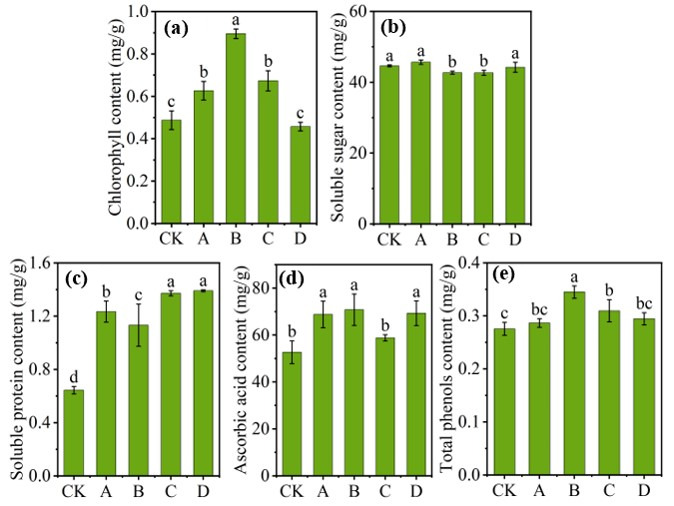
Chlorophyll contents (**a**), soluble sugar contents (**b**), soluble protein contents (**c**), ascorbic acid contents (**d**), and total phenol contents (**e**) of lettuce in different treatment groups. The same letters above the bars indicate no significant difference at the *p* ≤ 0.05 level.

To investigate the effects of SMS application on improving lettuce quality, we analyzed the chlorophyll, soluble sugar, soluble protein, ascorbic acid, and total phenolic contents in harvested lettuce leaves, and the results are presented in Figure 3.

Compared with the control group, lettuce grown in soil amended with SMS presented a higher chlorophyll content (Figure 3a), with Group B having the highest value (0.89 ± 0.02 mg g^−1^). In terms of soluble sugars (Figure 3b), the treatment groups differed from the control (44.63 ± 0.28 mg g^−1^) and were even lower, as seen in Group C (42.67 ± 0.71 mg g^−1^). In contrast, compared with the control group (0.64 ± 0.03 mg g^−1^), the soluble protein contents in Groups C and D were greater (1.37 ± 0.02 mg g^−1^ and 1.39 ± 0.01 mg g^−1^, respectively) (Figure 3c). Ascorbic acid (vitamin C), a common nutritional supplement that is beneficial to human health, was significantly enhanced by SMS and organic fertilizer application (Figure 3d). Group B achieved the highest concentration (70.77 ± 6.72 mg g^−1^). Total phenolics, another key bioactive compound in lettuce, were also higher in the treatment groups, peaking in Group B (0.34 ± 0.01 mg g^−1^). Therefore, SMS application effectively improved the nutritional quality of lettuce leaves, with an optimal dosage of 1.5 kg m^−2^ showing the most pronounced improvement.

### 3.4. Analysis of Soil Microbial Diversity

On the basis of the above findings, soil samples from Group B (SMS was 1.5 kg m^−2^), Group D (organic fertilizer addition), and the control group were collected after lettuce harvest for microbial community analyses. This investigation aimed to elucidate how SMS and organic fertilizer modulate soil microbiological properties, thereby providing mechanistic insights into the observed improvements in lettuce growth performance.

#### 3.4.1. Alpha Diversity

Alpha diversity reflects the species richness and evenness of a microbial community. The Chao 1, Shannon, Good’s coverage and Pielou_e indices represented the microbial richness, diversity, species coverage and community evenness, respectively.

**Figure 4:**
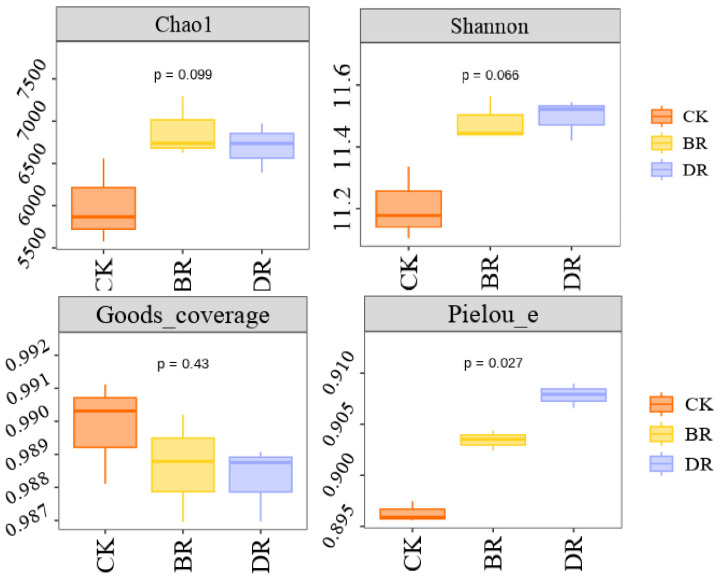
Effects of a 1.5 kg m^−2^ SMS addition (BR) and organic fertilizer treatment (DR) on the alpha diversity (e.g., Chao 1, Shannon, Goods_coverage, and Pielou_e indices) of soil bacteria; the control group was the CK group.

As shown in Figure 4, the Chao 1 diversity index, Shannon diversity index and Good’s coverage index had no significant differences among the control group and the Groups B and D (*p* = 0.099, *p* = 0.066, and *p*= 0.43, respectively; via the Student’s *t* test). while the Pielou_e index in the control group was lower than in Groups B and D (*p* = 0.027). Thus, SMS and organic matter addition improved the bacterial community evenness in the soil, but had little impact on microbial abundance and richness.

#### 3.4.2. Genus Level Analysis of the Soil Microbiome

**Figure 5:**
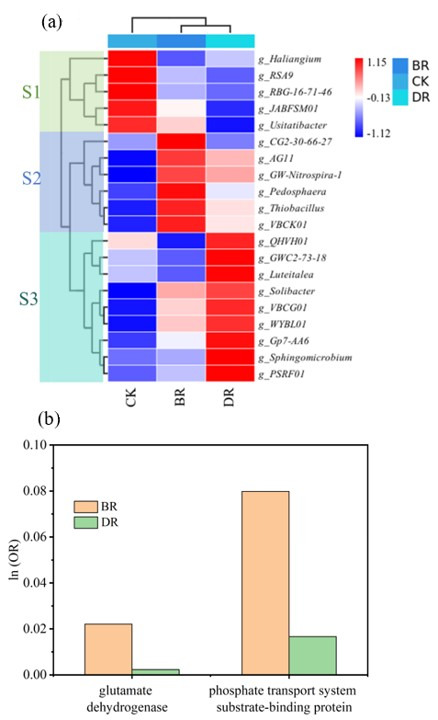
(**a**) Heatmap of 20 key bacterial genera that occupied ≥ 1% of at least one sample. The horizontal and vertical axes are stages and genera, respectively. The abundances of different species in the samples are displayed through the color gradient shown in the color column on the right side of the figure; its units are OTUs. (**b**) The odds ratios for enzyme-based functions for the BR and DR treatments compared with those for the control group (CK).

The results of the cluster analysis for the soil microbial communities (genus level) from the control (CK), B (BR), and D (DR) groups are shown in Figure 5. The figure also presents the abundances of individual microorganisms in the soil samples. The proportions of individual genera and the dominant genera in every sample are shown in Figure S2a. According to the genus-clustering tree (Figure 5), the genera could be divided into three groups: S1, which covered 10.45%, 6.13% and 5.13% of the CK, BR and DR treatments, respectively; S2, which occupied 5.81%, 13.07%, and 11.66% of the CK, BR and DR treatments, respectively; and S3, which covered 9.64%, 12.09% and 17.06% of the CK, BR and DR treatments, respectively, as shown in Figure S2b.

The abundances of all the S1 microbial consortia were greatest in the CK treatment, were decreased in the BR and DR treatments, and were lowest in the DR treatment. The genus *Haliangium* was reported to promote the synthesis of proteins and other substances in plants [27]. *RBG-16-71-46* belongs to a novel Thermoplasmata order and can undergo aerobic respiration to utilize and degrade complex carbon sources, including starch and glycogen [28]. *Usitatibacter*, belonging to the order Nitrosomonadales, possesses the gene *nosZ*, which encodes an enzyme that catalyzes the reduction of nitrous oxide to nitrogen and plays an important role in soil nitrogen cycling, particularly in denitrification [29,30].

The genera in the S2 Group demonstrated the highest relative abundances within the BR treatment. The genus *Nitrospira*, a widely distributed chemolithoautotrophic bacterium in soils, derives energy by oxidizing ammonia to nitrite and nitrate while simultaneously facilitating ammonia nitrogen fixation [31,32]. The genus *Pedosphaera* contains genes encoding cellulase, which is capable of degrading cellulose into β-glucose [33]. Furthermore, studies have reported that *Pedosphaera* produces glycoside hydrolases, which facilitate the breakdown of complex polysaccharides [34]. Therefore, the high relative abundance of *Pedosphaera* in the BR treatment can degrade SMSs in the soil and supply bioavailable carbon for bacterial growth. The genus *Thiobacillus*, a widely recognized denitrifying bacterium, contributes to soil nitrogen cycling by reducing nitrate to nitrite or even nitrogen gas (N_2_) [35]. Furthermore, this genus can solubilize soil phosphate minerals into plant-available phosphate (PO_4_^3−^), thereby increasing phosphorus uptake by plants. Concurrently, *Thiobacillus* assimilates a portion of the phosphate intracellularly, achieving microbial phosphorus immobilization and reducing phosphorus loss from the soil [36].

The relative abundances of genera in Group S3 were significantly greater in the DR Group. The genus *Luteitalea* is enriched in the *glpC* gene, which encodes an organophosphorus-degrading enzyme capable of converting organic phosphorus into inorganic phosphorus in the environment [37]. Some genera, such as *Solibacter* and *Sphingomicrobium*, are functionally important for soil nutrient retention and biogeochemical cycling. Specifically, *Solibacter* is involved in the fixation of inorganic nitrogen, whereas *Sphingomicrobium* increases the mobilization and cycling of key nutrients, such as nitrogen (N) and phosphorus (P) [37,38,39].

Table S1 listed the enzymes expected from the PICRUSt metagenomic investigation of the fates of nitrogen and phosphorus in the soil. The odds ratios for these enzyme-based functions for the BR and DR treatments compared with those for the control group (CK) are shown in Figure 5b. Glutamate dehydrogenase (GDH) is a key microbial enzyme that catalyzes the incorporation of ammonium into amino acids during nitrogen assimilation [40]. Compared with the control group, both the BR and DR groups presented significantly higher GDH levels, with the highest abundance observed in the BR treatment group. The same trend was also observed for the relative contents of phosphate transport system substrate-binding protein, which plays a crucial role in maintaining phosphorus balance in microorganisms [41]. Although the PICRUSt metagenomic investigation is not entirely precise, to a certain extent, it illuminated many issues. Therefore, these findings demonstrated that the application of SMS enhances the capacity of microbial nitrogen and phosphorus utilization.

#### 3.4.3. Relationships Among Soil Microbial Communities and Environmental Factors as Well as Lettuce Biochemical Properties

**Figure 6:**
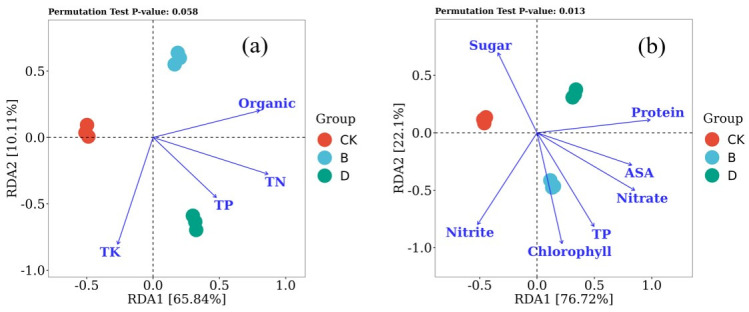
Redundancy analysis (RDA) of environmental factors (**a**) and lettuce biochemical properties (**b**) with respect to the microbial community abundances in the control group and in the B and D groups. The arrow lengths represent the magnitudes of the correlations between the factors and the bacterial community structures. The arrow directions represent the variation tendencies of the factors. ‘TP’ represents total phosphorus and total phenols in Figure 6a,b, respectively.

The relationships between the environmental factors and the microbial community abundances in the control group and the B and D groups are shown in Figure 6a. The first axis explained 65.84%, and the second axis explained 10.11% of the variation in microbial diversity. Total potassium (TK) and organic content were clearly negatively correlated, whereas the organic content was positively correlated with total nitrogen (TN) and total phosphorus (TP). In the control group, the microbial community was influenced mainly by TK, whereas in Groups B and D, they were influenced primarily by the organic and total phosphorus contents, respectively. The relationships between the lettuce biochemical properties and the microbial community abundances are shown in Figure 6b. The first and second axes explained 76.43% and 21.94%, respectively, of the variation in microbial diversity. Sugars were significantly negatively correlated with other biochemical properties (e.g., chlorophyll, total phenols, ASA, and protein), whereas chlorophyll and total phenols were strongly positively correlated. In Group B, all the properties except sugar were positively correlated with the microbial communities, whereas in the control group (CK), the opposite pattern was observed: sugar was positively correlated, but the other components were negatively correlated. These results suggested that the application of SMS not only adds nutrients to the soil, improves the soil environment, and affects the growth of lettuce but also may affect the quality of lettuce by influencing changes in the soil microbial community.

### 3.5. Economic Costs Required for Increasing Lettuce Weight

**Figure 7:**
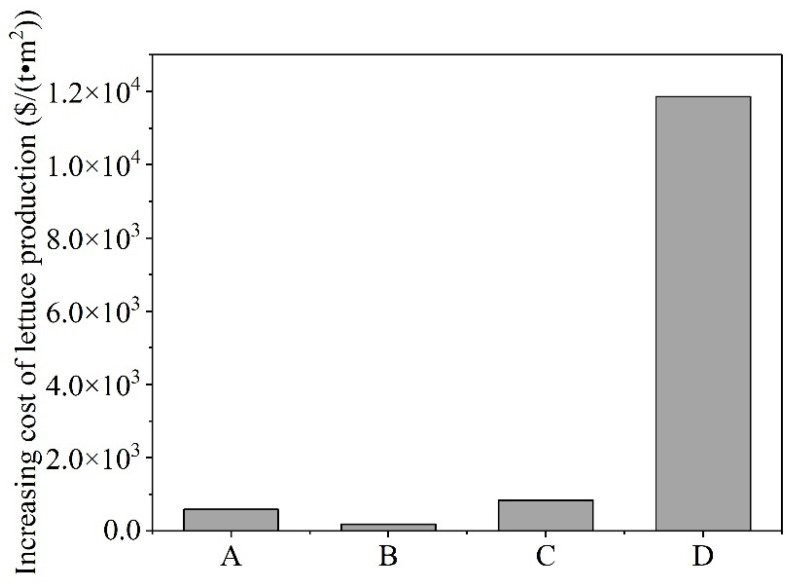
The economic costs required for lettuce increased compared with those of the control group.

The increasing economic costs of applying SMS and organic fertilizer for lettuce were calculated, and the results are shown in Figure 7. Compared with that in the control group, for every additional metric ton of lettuce yield, the fertilizer costs in the other groups (e.g., A, B, C and D) increased by 583, 178, 83.81 and 11,858 $ per square meter per metric ton, respectively. Therefore, the application of 1.5 kg m^−2^ of composted SMS resulted in the lowest additional cost and the highest economic return.

## 4. Conclusions

The addition of SMS and organic fertilizer increased the contents of total nitrogen, total phosphorus, and potassium in the soil but also promoted the growth and quality of the lettuce and improved the contents of chlorophyll and total phenols. The best promoting effect was achieved when the SMS application rate was 1.5 kg m^−2^, notably increasing the organic matter content by 27.9%, enhancing the average weight of lettuce by 71.7%, and boosting the levels of chlorophyll and total phenols by 45.6% and 25.2%, respectively. Soil microbial diversity analysis revealed that both SMS and organic fertilizer improved the soil microbial diversity. Microbial community analyses indicated that the dominant microbial communities varied among the different treatment groups. Interestingly, although the addition of SMS and organic fertilizer improved the efficiency of the microbial conversion of nitrogen and phosphorus compared with that in the control group, the improvement in the efficiency of SMS was greater, and the cost of SMS was lower. Thus, returning SMS to the field not only solves the problem of SMS treatment and disposal but also improves the soil environment and enhances the quality of agricultural products. Establishing a scientific technical pathway for returning *Volvariella volvacea* SMS to the field is the next step for its widespread application.

## Data Availability

The original data presented in the study are openly available in NCBI under the access number is PRJNA1414867.

## Supplementary Materials

The following supporting information can be downloaded at: https://www.mdpi.com/article/10.3390/microorganisms14050985/s1, Figure S1: Distribution of experimental fields.; Figure S2: Phylogenetic structure of the soil samples at the genus level (a), and the sum of genera abundance in each group (b). Table S1: Enzymes involved in nitrogen and phosphorus based on PICRUSt metagenomic predictions. File S1: The economic costs calculation for lettuce increased.

## References

[1] Stojanović M., Petrović I., Žuža M., Jovanović Z., Moravčević Đ., Cvijanović G., Savić S. (2020). The Productivity and Quality of *Lactuca sativa* as Influenced by Microbiological Fertilisers and Seasonal Conditions. Zemdirbyste-Agriculture.

[2] Zhou W., Liang X., Dai P., Chen Y., Zhang Y., Zhang M., Lu L., Jin C., Lin X. (2019). Alteration of Phenolic Composition in Lettuce (*Lactuca sativa* L.) by Reducing Nitrogen Supply Enhances Its Anti-Proliferative Effects on Colorectal Cancer Cells. Int. J. Mol. Sci..

[3] Lallukka T., Hiilamo A., Pietiläinen O., Mänty M., Kouvonen A., Rahkonen O. (2020). Who Maintains Good Health Functioning? The Contribution of Social, Work-Related and Behavioural Factors to Mental and Physical Health Functioning Trajectories in Ageing Employees. Occup. Environ. Med..

[4] Kim M.J., Moon Y., Tou J.C., Mou B., Waterland N.L. (2016). Nutritional Value, Bioactive Compounds and Health Benefits of Lettuce (*Lactuca sativa* L.). J. Food Compos. Anal..

[5] Dong M., Zhou H., Wang J., Yang J., Lai J., Chen Y., Sun F., Ye X., Wu Y. (2024). Responses of Soil Microbial Metabolism, Function and Soil Quality to Long-Term Addition of Organic Materials with Different Carbon Sources. Biochar.

[6] Duan X., Rui Y., Xia Y., Hu Y., Ma C., Qiao H., Zeng G., Su Y., Wu J., Chen X. (2024). Higher Microbial C Use Efficiency in Paddy than in Adjacent Upland Soils: Evidence from Continental Scale. Soil Tillage Res..

[7] Li P., Kong D., Zhang H., Xu L., Li C., Wu M., Jiao J., Li D., Xu L., Li H. (2021). Different Regulation of Soil Structure and Resource Chemistry under Animal- and Plant-Derived Organic Fertilizers Changed Soil Bacterial Communities. Appl. Soil Ecol..

[8] Tao R., Wakelin S.A., Liang Y., Hu B., Chu G. (2018). Nitrous Oxide Emission and Denitrifier Communities in Drip-Irrigated Calcareous Soil as Affected by Chemical and Organic Fertilizers. Sci. Total Environ..

[9] Polthanee A., Tre-loges V., Promsena K. (2008). Effect of Rice Straw Management and Organic Fertilizer Application on Growth and Yield of Dry Direct-Seeded Rice. Paddy Water Environ..

[10] Li F., Kong Q., Zhang Q., Wang H., Wang L., Luo T. (2020). Spent Mushroom Substrates Affect Soil Humus Composition, Microbial Biomass and Functional Diversity in Paddy Fields. Appl. Soil Ecol..

[11] Kakumyan P., Yang L., Liu S., Saninjuk K., Dong Q., Pan X., Yu C., Zhao Y. (2025). Sustainable Recycling of Mushroom Residue as an Effective Substitute for Cotton Hull Waste in *Volvariella volvacea* Cultivation: Evidence from Physicochemical and Microbiome Analyses. Microorganisms.

[12] Duan Y., Wang M., Wang L., Wu G., Mao T., Sun H., Pang H., Zhang M., Jiao Z., Wang Y. (2024). Mushroom Residue and Sheep Manure Fermentation with *Bacillus* Promoted Tomato Growth via Nutrient Release and Favorable Microbial Conditions. Chem. Biol. Technol. Agric..

[13] Lian J., Qu L., Ren P., Ren H., Wan L., Guo H., Zhang H., Chang S., Gao X., Ban L. (2021). Industrial Mushroom Residue as Cow Bedding: Analysis of Microbial Diversity and Applications. Curr. Microbiol..

[14] Qiao Y., Tie J., Wang X., Wei B., Zhang W., Liu Z., Zhang G., Lyu J., Liao W., Hu L. (2023). Comprehensive Evaluation on Effect of Planting and Breeding Waste Composts on the Yield, Nutrient Utilization, and Soil Environment of Baby Cabbage. J. Environ. Manag..

[15] Tang Q., Liu W., Huang H., Peng Z., Deng L. (2024). Responses of Crop Yield, Soil Fertility, and Heavy Metals to Spent Mushroom Residues Application. Plants.

[16] Alves L.D.S., Caitano C.E.C., Ferrari S., Vieira Júnior W.G., Heinrichs R., De Almeida Moreira B.R., Pardo-Giménez A., Zied D.C. (2022). Application of Spent Sun Mushroom Substrate in Substitution of Synthetic Fertilizers at Maize Topdressing. Agronomy.

[17] Muchena F.B., Pisa C., Mutetwa M., Govera C., Ngezimana W. (2021). Effect of Spent Button Mushroom Substrate on Yield and Quality of Baby Spinach (*Spinacia oleracea*). Int. J. Agron..

[18] Demir H., Saka A.K., Uçan U., Akgün İ.H., Yalçı H.K. (2024). Impact of Effective Micro-Organisms (EM) on the Yield, Growth and Bio-Chemical Properties of Lettuce When Applied to Soil and Leaves. BMC Plant Biol..

[19] Zengin F. (2013). Physiological Behavior of Bean (*Phaseolus vulgaris* L.) Seedlings under Metal Stress. Biol. Res..

[20] Zhang Y., Cheng W., Di H., Yang S., Tian Y., Tong Y., Huang H., Escalona V.H., Tang Y., Li H. (2024). Variation in Nutritional Components and Antioxidant Capacity of Different Cultivars and Organs of Basella Alba. Plants.

[21] Xiao C., Sun F. (2013). Fabrication of Distilled Water-Soluble Chitosan/Alginate Functional Multilayer Composite Microspheres. Carbohydr. Polym..

[22] Chao Y.-Y., Hong C.-Y., Kao C.H. (2010). The Decline in Ascorbic Acid Content Is Associated with Cadmium Toxicity of Rice Seedlings. Plant Physiol. Biochem..

[23] Keskin L., Turkmen O., Paksoy M., Keskin L., Yüceol F., Çınar N., Çınar O., Hacıküçük A., Namal H., Göktürk R.S. (2023). Determination of Some Morphological Traits and Total Phenolic and Flavonoid Contents in *Cynara* spp. Collected from Turkey and North Cyprus Turkish Republic. J. Elem..

[24] Li Q., Zhang D., Song Z., Ren L., Jin X., Fang W., Yan D., Li Y., Wang Q., Cao A. (2022). Organic Fertilizer Activates Soil Beneficial Microorganisms to Promote Strawberry Growth and Soil Health after Fumigation. Environ. Pollut..

[25] Ontiveros-Valencia A., Zhou C., Ilhan Z.E., De Saint Cyr L.C., Krajmalnik-Brown R., Rittmann B.E. (2017). Total Electron Acceptor Loading and Composition Affect Hexavalent Uranium Reduction and Microbial Community Structure in a Membrane Biofilm Reactor. Water Res..

[26] Medina E., Paredes C., Bustamante M.A., Moral R., Moreno-Caselles J. (2012). Relationships between Soil Physico-Chemical, Chemical and Biological Properties in a Soil Amended with Spent Mushroom Substrate. Geoderma.

[27] Lin W., Liu L., Liang J., Tang X., Shi J., Zhang L., Wu P., Lan S., Wang S., Zhou Y. (2022). Changes of Endophytic Microbial Community in *Rhododendron simsii* Roots under Heat Stress and Its Correlation with Leaf Physiological Indicators. Front. Microbiol..

[28] Yuan Y., Liu J., Yang T.-T., Gao S.-M., Liao B., Huang L.-N. (2021). Genomic Insights into the Ecological Role and Evolution of a Novel *Thermoplasmata* Order, “*Candidatus* Sysuiplasmatales”. Appl. Environ. Microbiol..

[29] Fernández-López M.G., Sánchez-Reyes A., Rosas-Ramírez M.E., Balcázar-López E. (2024). Microbiodiversity Landscape Present in the Mine-Tailings of the “*Sierra de Huautla*” Biosphere Reserve, Mexico. Water Air Soil Pollut..

[30] Mise K., Masuda Y., Senoo K., Itoh H. (2025). Betaproteobacterial Clade II *nosZ* Activated under High N_2_O Concentrations in Paddy Soil Microcosms. J. Appl. Microbiol..

[31] Hu J., Zhao Y., Yao X., Wang J., Zheng P., Xi C., Hu B. (2021). Dominance of Comammox *Nitrospira* in Soil Nitrification. Sci. Total Environ..

[32] Xu S., Wang B., Li Y., Jiang D., Zhou Y., Ding A., Zong Y., Ling X., Zhang S., Lu H. (2020). Ubiquity, Diversity, and Activity of Comammox *Nitrospira* in Agricultural Soils. Sci. Total Environ..

[33] Zhang X., Zhong Z., Bian F., Yang C. (2019). Effects of Composted Bamboo Residue Amendments on Soil Microbial Communities in an Intensively Managed Bamboo (*Phyllostachys praecox*) Plantation. Appl. Soil Ecol..

[34] Dang H., Yu N., Mou A., Zhang L., Guo B., Liu Y. (2022). Metagenomic Insights into Direct Interspecies Electron Transfer and Quorum Sensing in Blackwater Anaerobic Digestion Reactors Supplemented with Granular Activated Carbon. Bioresour. Technol..

[35] Li L., Wang T., Sun Y., Wang P., Yvette B., Meng J., Li Q., Zhou Y. (2017). Identify Biosorption Effects of Thiobacillus towards Perfluorooctanoic Acid (PFOA): Pilot Study from Field to Laboratory. Chemosphere.

[36] Jazaeri M., Akhgar A., Sarcheshmehpour M., Mohammadi A.H. (2016). Bioresource Efficacy of Phosphate Rock, Sulfur, and *Thiobacillus* Inoculum in Improving Soil Phosphorus Availability. Commun. Soil Sci. Plant Anal..

[37] Cheng X., Qu M., Hu Y., Liu X., Mei Y. (2025). Differences in Microbial Communities and Phosphorus Cycles between Rural and Urban Lakes: Based on Glyphosate and AMPA Effects. J. Environ. Manag..

[38] Chen Y., Wang S., Li Y., Liu W., Niu Z. (2024). Response of Bacterial Community Structure and Function in Rhizosphere Soil on the Photosynthesis of Selected Plant Types C3 and C4 under Bis(2,4,6-Tribromophenoxy) Ethane Exposure. Agriculture.

[39] Hirpara K.R., Hinsu A.T., Kothari R.K. (2024). Metagenomic Evaluation of Peanut Rhizosphere Microbiome from the Farms of Saurashtra Regions of Gujarat, India. Sci. Rep..

[40] Peralta G.H., Bergamini C.V., Hynes E.R. (2016). Aminotransferase and Glutamate Dehydrogenase Activities in *Lactobacilli* and *Streptococci*. Braz. J. Microbiol..

[41] Baz L., Abulfaraj A.A., Tashkandi M.A., Baeissa H.M., Refai M.Y., Barqawi A.A., Shami A., Abuauf H.W., Ashy R.A., Jalal R.S. (2022). Predicted Functional Shifts Due to Type of Soil Microbiome and Watering of Two Wild Plants in Western Region of Saudi Arabia. Phyton.

