# Supplementary material for: Spent Mushroom Substrate Reused as Organic Fertilizer Enhances Lettuce (Lactuca sativa L.) Quality and Soil Nutrients: Insights from Physicochemical and Microbiome Analyses
Source: Microorganisms. 2026 Apr 28;14(5):985. doi: 10.3390/microorganisms14050985 (PMC13209306; doi:10.3390/microorganisms14050985)
Supplement: Supplementary file 1 [file microorganisms-14-00985-s001.zip › microorganisms-4148641-supplementary.pdf]

Supplementary Information

Spent Mushroom Substrate Reused as Organic Fertilizer Enhances Lettuce (*Lactuca sativa* L.) Quality and Soil Nutrients: Insights from Physicochemical and Microbiome Analyses

Lin Yang [1], Zhengpeng Li [1], Shiwei Wei [2], Qin Dong [1], Lei Zha [1], Changxia Yu [1,*] and Yan Zhao [1,*]

1 Institute of Edible Fungi, Shanghai Academy of Agricultural Sciences, Shanghai 201403, China; ylin_jade@163.com (L.Y.); lizp_ln@126.com (Z.L.); maomao88719@163.com (Q.D.); zhalei@saas.sh.cn (L.Z.)

2 Shanghai Agrobiological Gene Center, Shanghai 201106, China; wsw@sagc.org.cn

* Correspondence: ycx41529@163.com (C.Y.); jiandan289@126.com (Y.Z.)

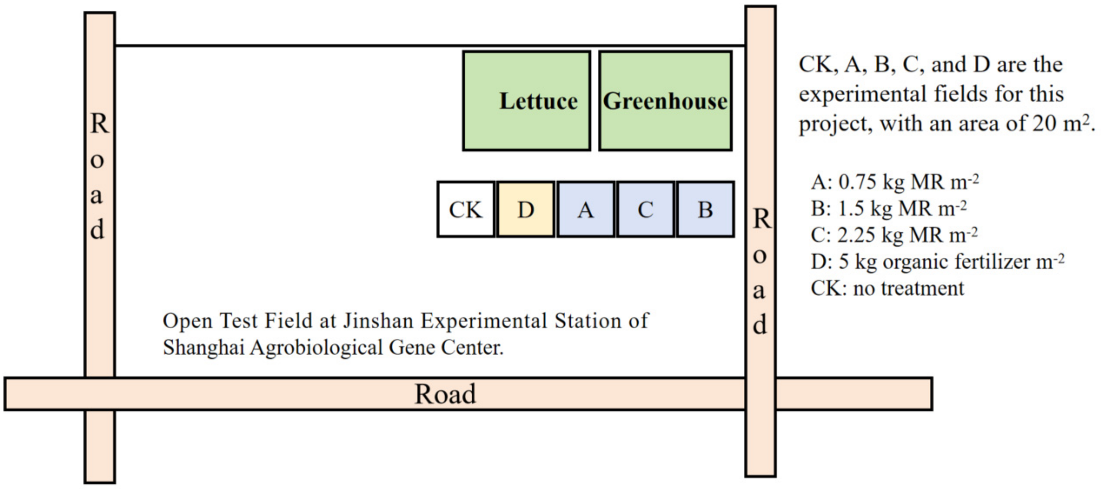

Figure S1. Distribution of experimental fields.

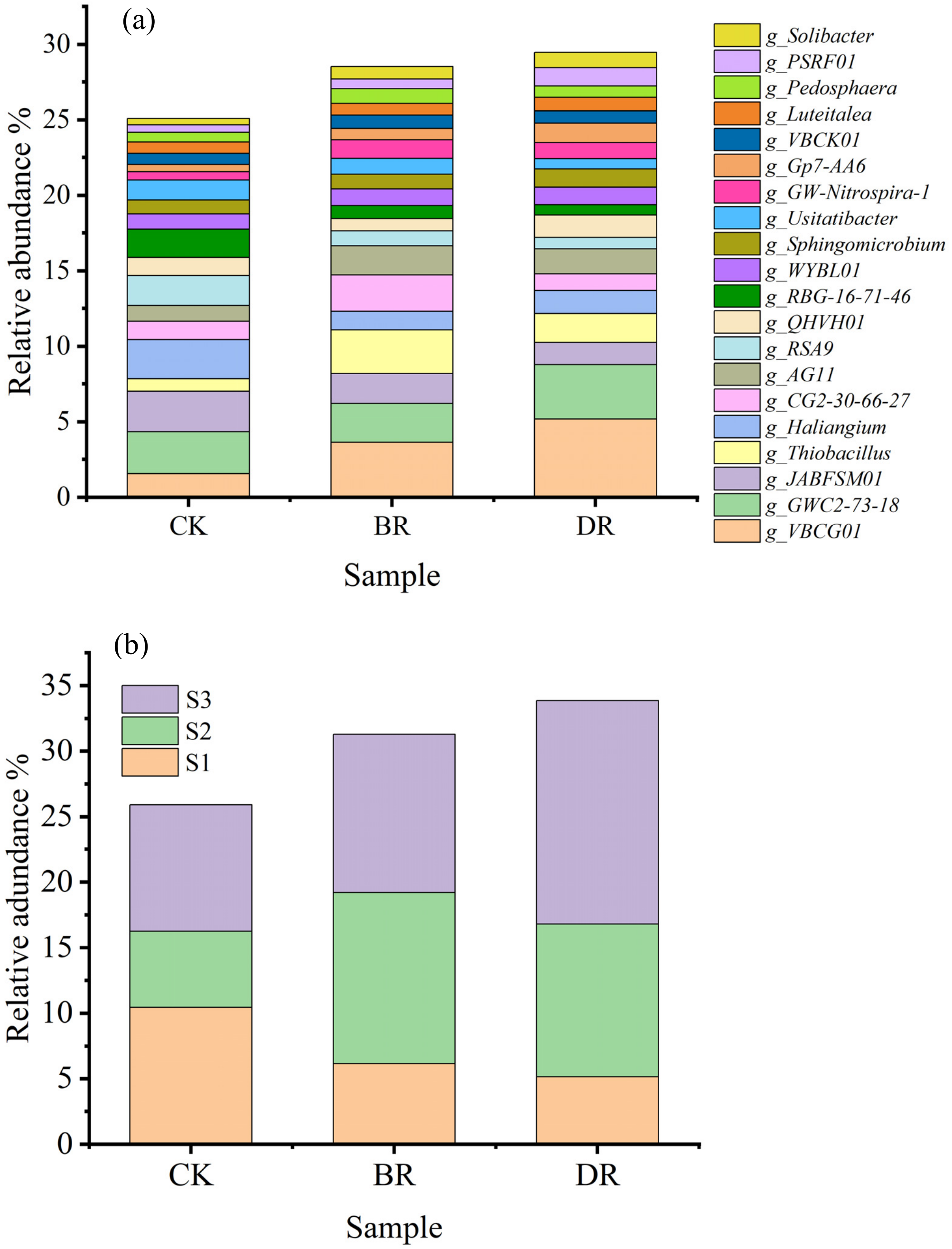

Figure S2. Phylogenetic structure of the soil samples at the genus level (a), and the sum of genera abundance in each group (b).

Table S1. Enzymes involved in nitrogen and phosphorus based on PICRUSt metagenomic predictions.

Nutrient convey	KO	Description	Control group 1	Control group 1	Control group 1	B1	B2	B3	D1	D2	D3
nitrogen	K00260	glutamate dehydrogenase	0.01066	0.01063	0.01062	0.01085	0.01087	0.01091	0.01064	0.01069	0.01066
phosphorus	K02040	phosphate transport system substrate-binding protein	0.01035	0.01019	0.01019	0.01105	0.01107	0.01113	0.01035	0.01049	0.01040
